# Using Point-of-Choice Prompts to Reduce Sedentary Behavior in Sit-Stand Workstation Users

**DOI:** 10.3389/fpubh.2018.00323

**Published:** 2018-11-21

**Authors:** Miranda L. Larouche, Sarah L. Mullane, Meynard John L. Toledo, Mark A. Pereira, Jennifer L. Huberty, Barbara E. Ainsworth, Matthew P. Buman

**Affiliations:** ^1^College of Health Solutions, Arizona State University, Phoenix, AZ, United States; ^2^Division of Epidemiology and Community Health, University of Minnesota, Minneapolis, MN, United States

**Keywords:** office workers, sedentary behavior, intervention, preference, acceptability, efficacy

## Abstract

**Introduction:** Desk-based office workers are at occupational risk for poor health outcomes from excessive time spent sitting. Sit-stand workstations are used to mitigate sitting, but lack of workstation usage has been observed. Point-of-choice (PoC) prompts offer a complementary strategy for office workers to break up their sitting time.

**Study purpose:** The purpose of this study was to examine the preliminary efficacy, preference, and acceptability of a theory-driven (i.e., 40 unique prompts encompassing social cognitive theory; TD-PoC) and an atheoretical basic reminder PoC prompt intervention (R-PoC) on reducing sedentary behavior in office workers with self-reported low sit-stand workstation usage (≤4 h per day).

**Methods:** In a cross-over design, participants (*N* = 19, 78.9% female, 39.4 ± 10.7 years of age) completed a 5-days no-prompt control condition followed by a random and counterbalanced assignment to one of the TD-PoC and R-PoC active conditions with a 1-week washout period between. Preliminary efficacy was assessed during work hours with the activPAL micro accelerometer. Preference was assessed prior to each active condition and acceptability was assessed following each active condition via questionnaire.

**Results:** The R-PoC prompt condition significantly decreased sitting time (b[se] = −49.0 [20.8], *p* = 0.03) and increased standing time (b[se] = 49.8 [19.7], *p* = 0.02) and displayed a significant increase in sit-stand transitions (b[se] = 2.3 [1.1], *p* = 0.04), relative to no-prompt control. Both the R-PoC and TD-PoC prompt conditions significantly decreased time spent in prolonged sitting bouts at b[se] = −68.1 [27.8], (*p* = 0.02), (b[se] = −76.7 [27.1], *p* = 0.008) relative to no-prompt control. Overall, the TD-PoC prompt condition displayed higher preference and acceptability ratings; however, these differences were not significant (*p*'s > 0.05).

**Conclusion:** While the R-PoC prompt condition was slightly more efficacious than the TD-PoC prompt condition, the TD-PoC prompt condition was rated with higher preference and acceptability scores. Large variations between participants in preference, acceptability, and intervention feedback may indicate need for tailored messaging which may facilitate sustained use in the long-term.

## Introduction

Desk-based occupations confine office workers to a seated position during the working day (1) of which 70–80% of work time is spent seated (2). Sedentary behaviors (i.e., waking behaviors in a seated or reclining posture <1.5 metabolic equivalents [METs]) (3) are associated with deleterious health outcomes, including an increased risk for cardiometabolic disease and early mortality (4–8). In particular, prolonged sedentary behavior, without standing or light-intensity physical activity (LPA), acutely and negatively impacts circulating blood glucose (4, 5), blood pressure (8) and musculoskeletal pain (9). Regularly interrupting sedentary time with bouts of standing (light-physical activity i.e., LPA have been shown to reduce these effects (4, 5, 8, 10–15). Therefore, an increasingly popular strategy to reduce workplace sedentary behavior is the use of sit-stand workstations, which provide desk-based office workers with the opportunity to alternate between seated and standing positions throughout the day (16). Behavioral trials incorporating sit-stand workstations have demonstrated significant reductions in workplace sitting and these effects appear sustained over 18 months (17). However, avoiding prolonged bouts of sitting (18) and sustaining frequent sit-stand workstation use over time continues to be a challenge due to the habitual nature of sitting in the workplace (19).

Point-of-choice (PoC) prompts, also known as point-of-decision prompts, are an effective tool for encouraging individuals to make more active decisions over sedentary ones (20). To date, PoC prompts have largely focused on environmental components such as stair use and have been shown to be effective for increasing physical activity (21). PoC prompts have been delivered in the workplace to interrupt prolonged sitting using visual cues, such as signage (20, 22), email reminders (23), computer software (24), and more recently, wearable devices (25). One PoC prompt study in the workplace found that prompting desk-based office workers to stand for one minute every 30 min, using computer software installed on their work computer, was effective for reducing total sitting and prolonged bouts of sitting (26). However, both the PoC prompt intervention and control group received educational benefits of reduced prolonged sitting time at the start of the study, making it difficult to conclude if findings were a direct result of prompts, or reinforcement from education. Similarly, Donath et al. (22) used computer-based PoC prompts (three times daily over 12 weeks) among those with sit-stand workstations and found marginal reductions in sedentary time via increased standing. In contrast to the prompts used by Evans et al. (26)—which simply reminded participants to take a break—the prompts used by Donath et al. (22) consisted of three different reminders, one of which indicated that “prolonged sitting is harmful” (i.e., outcome expectancies). Furthermore, in both studies, neither preference (i.e., patient choice of given characteristics of an intervention or treatment), which provides insight for intervention adoption (27), or acceptability [i.e., attitude toward the intervention or treatment options considering characteristics of the intervention; (28)] of the prompt conditions was fully assessed. Preference and acceptability are necessary constructs to assess and enhance user adherence to a treatment, satisfaction, and the validity of research. Additionally, preference and acceptability help to inform interventions and improve outcomes (28).

PoC prompts may be an effective complementary strategy to sit-stand workstation use. However, there is limited understanding regarding the effects of prompt content in this context. Previous workplace PoC prompts have been atheoretical in nature (22, 26), yet there is evidence to suggest that interventions derived from a theoretical framework may be more effective (29) and are likely to lead to more sustained behavior change. Large scale studies targeting sedentary behavior in the workplace have utilized social cognitive theory (SCT) in the development of their intervention strategies (11, 30). The framework of the SCT is relevant to the workplace environment due its triadic, reciprocal nature in which considerations are given to interactions among the environment, individual, and behavior (31). Key components of the SCT that may be efficacious for enhancing sit-stand workstation utilization include: (a) the development of self-efficacy (i.e., level of confidence in ability to exercise control over a behavior) for using the workstation; and (b) outcome expectations (i.e., individual perceptions that a given behavior will result in an outcome) for establishing benefits for engaging in standing and (c) proximal goal-setting (i.e., intentions) for success (31). Research has yet to investigate the integration of the SCT into point-of-choice prompts within the workplace. We postulate that the by integrating the SCT into a point-of-choice prompt message, behavior change may be facilitated by aiding individuals abilities to cope with barriers hindering behavior change and fostering self-efficacy and mastery to break-up their sitting time throughout their working day. As such, creating brief, unique prompts encompassing these constructs may help to facilitate proficiency in the behavior and promote long-term sustained behavior change.

The primary aim of our study was to examine the preliminary efficacy, preference, and acceptability, of two workplace PoC prompt interventions (i.e., atheoretical basic reminder [R-PoC] vs. theory-driven [TD-PoC]) for reducing sedentary time in office workers with suboptimal compliance for sit-stand workstation usage. We hypothesized prolonged sitting time at work would be reduced for both intervention conditions relative to the no-prompt control condition, and that the TD-PoC prompt condition would achieve greater reductions than that of the R-PoC prompt condition. We also hypothesized that participants would prefer and find more acceptable the TD-PoC prompt condition relative to the R-PoC prompt condition.

## Materials and methods

### Study sample

Participants were desk-based office workers who currently have a sit-stand workstation installed at their primary office space but reported suboptimal utilization. Participants were recruited across the Phoenix metropolitan area using an information flier at the Arizona State University (ASU) Tempe and Phoenix campuses as well as several medium and large worksites. Inclusion criteria for the study were: age 18 years and older, full-time employee (>30 h/week), in office at least four days per week, in a seated position for majority of working day, had a sit-stand workstation installed at primary desk, reported using sit-stand workstation ≤4 h of the working day, and able and willing to engage in study assessment and intervention for 4 weeks. Exclusion criteria were: non-English speaking, advised by a health professional to avoid long periods of standing, and pregnant women entering or in the third trimester. All participants provided informed consent prior to participation and this study was approval by the ASU institution review board.

### Study design and procedures

**Figure 1:**
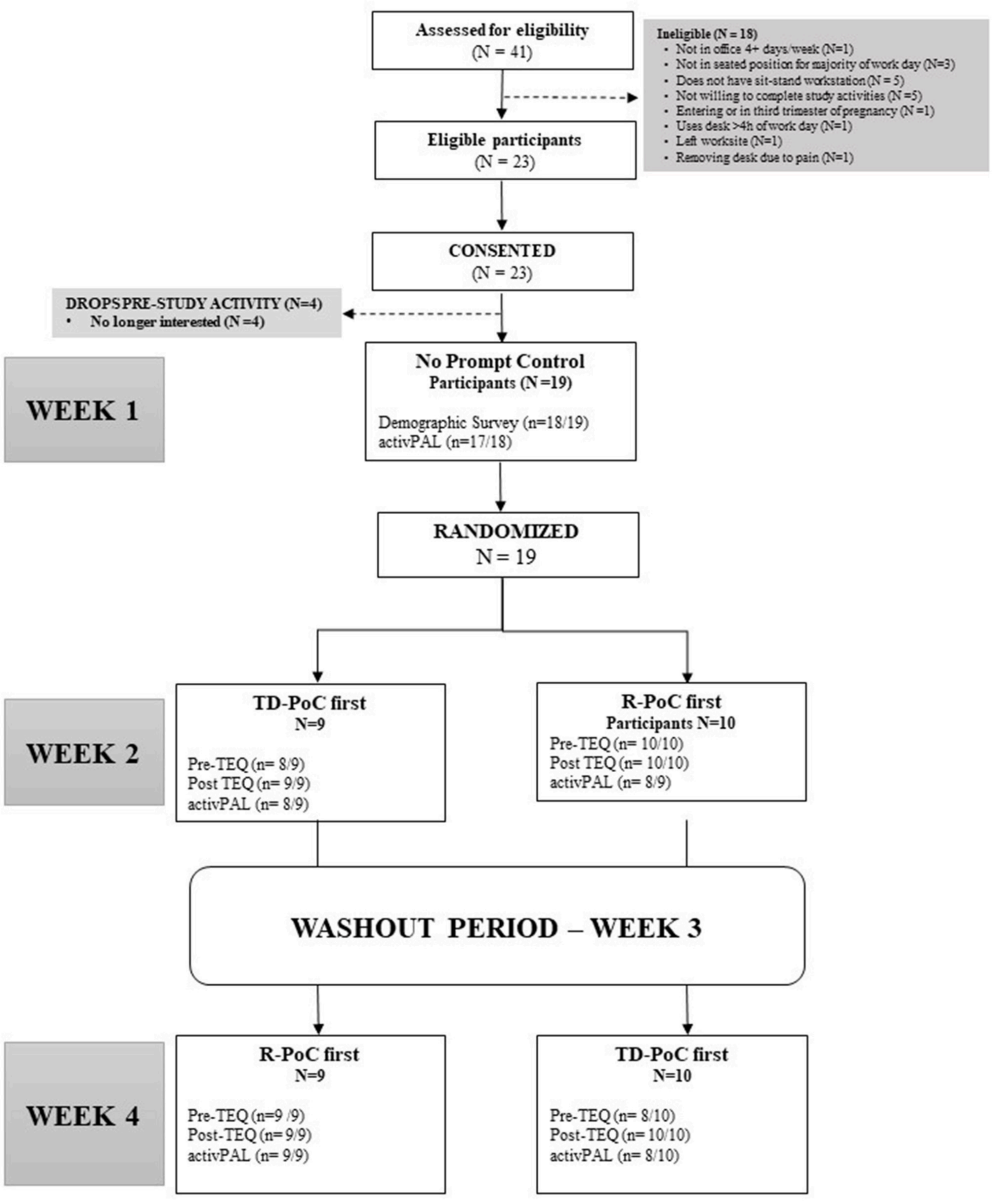
Consort diagram.

Figure 1 provides an overview of study design. We conducted a randomized cross-over trial. Enrollment and participation in this study took place from November 2017–March 2018. Total study participation lasted 30 days. Participants completed a no-prompt control condition for 5 work days to establish baseline sedentary time. Participants were then randomized to complete one of the active prompt conditions (i.e., R-PoC or TD-PoC) first. After completion of the first respective 5-day active condition, participants entered a 1-week washout period, in which they were not sent prompts and no assessment of sedentary time was collected. Participants finally entered their last 5-day active prompt condition in which they received the intervention they were not originally assigned.

### Prompt content and administration

As displayed in Table 1, the R-PoC prompt condition consisted of the administration of the same single prompt: “Time to STAND!” Whereas, the TD-PoC prompt condition consisted of the distribution of 40 unique prompts encompassing SCT constructs (i.e., self-efficacy, outcome expectancies, and proximal goal setting). For both the active prompt conditions, prompts were sent eight times/day to participant emails using the web-based platform, MailChimp (Email marketing company, Atlanta, GA). These email prompts (i.e., <50 words on subject line of an email) were sent between the hours of 9:00 am and 6:00 pm with the lunch hour (i.e., 12:00 a.m.−1:00 p.m.) being avoided. Furthermore, the time within each hour (i.e., on the hour, or 15 min, 30 min, or 45 min past) each prompt was sent was randomized to avoid anticipation of the prompt. Lastly, all participants followed the same random schedule during each active prompt condition.

**Table 1:** TD-PoC prompt content and schedule.

**DAY**	**AM/PM**	**TYPE**	**NUM**.	**PROMPT**	**Time**
DAY 1	AM	SE	1	SAY IT: I have the ability to STAND while I work	0
		OE	2	Did you know? STANDing can re-energize and maintain focus	45
		SE	3	SAY IT: I CAN use my sit-stand workstation to STAND and work	30
	PM	PG	4	GOAL: STAND while you email today	15
		SE	5	SAY IT: I can STAND while I work!	0
		OE	6	Can't concentrate? STAND to clear your mind!	45
		SE	7	SAY IT: I know I will Stand at work	30
		PG	8	GOAL: STAND when someone visits your desk	15
DAY 2	AM	SE	9	SAY IT: It is MY choice to STAND and work	30
		OE	10	Break away from sitting to clear your head – STAND	15
		SE	11	SAY IT: I am STANDing more at work	0
	PM	PG	12	GOAL: STAND when your phone rings	45
		SE	13	SAY IT: I will STAND and work	45
		OE	14	Engaged muscles = improved blood flow – STAND	15
		SE	15	SAY IT: I WILL use my sit-stand workstation today	0
		PG	16	GOAL: STAND when you transition between tasks	30
DAY 3	AM	SE	17	SAY IT: I WILL balance my sitting time by STANDing	15
		OE	18	Need energy - Take a STAND	45
		SE	19	SAY IT: I WILL accomplish my goal to STAND and work	0
	PM	PG	20	GOAL: STAND while reading	30
		SE	21	Keep STANDing, look at how far you've come!	15
		OE	22	STAND up - be good to yourself	0
		SE	23	You've made it this far, don't stop now! STAND!	45
		PG	24	GOAL: STAND while you problem solve	30
DAY 4	AM	SE	25	Keep it up! Beat your sitting habit, STAND!	45
		OE	26	Stop stressing about a deadline – STAND!	30
		SE	27	You're making progress, keep STANDing while you work!	0
	PM	PG	28	GOAL: STAND for the next 5-min	15
		SE	29	Fight back against sitting, take a STAND now!	0
		OE	30	Help yourself get a good night rest – STAND	15
		SE	31	The choice is yours, sit or STAND!	30
		PG	32	GOAL: STAND for the next 10-min	45
DAY 5	AM	SE	33	Keep it going you're still STANDing	30
		OE	34	Too much sitting = poor health outcomes, STAND!	15
		SE	35	Don't let setbacks halt your progress, STAND!	45
	PM	PG	36	GOAL: STAND for the next 15-min	0
		SE	37	Continue your successes now by STANDing	15
		OE	38	Reduce your risk for diabetes - STAND!	45
		SE	39	You have CAN STAND and work	30
		PG	40	GOAL: STAND for the next 30-min	0

## Measures

### Demographics

Age, gender, height, and weight were obtained during the no-prompt control condition via an electronic survey administered using Qualtrics (Software Company, UT).

### Preliminary efficacy of changes in workplace sedentary time

To objectively assess sedentary time during all study conditions, the activPAL micro accelerometer was worn during all working days. This device is valid and reliable (32–34). We derived the following measures from the activPAL: sitting, standing, stepping, and sitting bouts >30 min, and sit-stand transitions per sedentary hour. Appropriate time-based variables were standardized to an 8-h workday. The activPAL device was waterproofed and participants were instructed to affix the device to the midline of their right thigh using hypoallergenic tape (i.e., Hypafix) and wear for five consecutive working days during the no-prompt control and active prompt conditions. Data collected from this device were processed into events of sitting, standing, or, stepping using the activPAL software (activPAL version 7.2.32 PAL Technologies Ltd, Scotland, UK). Workday arrival and departure were self-reported using a paper daily log. Any periods of continuous sitting or standing behavior >6 h as indicated by the activPAL (i.e., non-wear time) were excluded from the analyses. Wear time of at least three days with >4 h of work time was required for inclusion.

### Preference of PoC prompt conditions

To assess preference for each PoC prompt condition, we adapted the Therapy Evaluation Questionnaire (TEQ) from the Treatment Acceptability and Preferences (TAP) measure, a valid tool for measuring preference (35). We administered the Pre-TEQ prior to each active condition to assess participant preference (i.e., preference rating for each TEQ item with a brief description of the respective condition [repetitive reminder prompt vs. unique theory-driven prompts]) of each PoC prompt intervention. The Pre-TEQ used in the present study included eight items on a 5-point Likert scale (Not at all [0] to Totally [4]). The items assessed the following: how logical the prompts seemed; how easy it would be to respond to prompts; how appropriate the prompts would be for standing; how helpful prompts would be for standing; how successful they believe the prompts would be for managing standing; how confident they were that they could stand in response to prompts; how likely they were to recommend the prompt intervention; and how important it was to make prompts available to others with a sit-stand workstation. Consistent with previous research (36, 37) we established a benchmark of ≥ 70% preference (defined as a rating of “Very” or “Totally”) as a criterion of success.

### Acceptability of PoC prompt conditions

To assess acceptability (i.e., acceptability rating for each TEQ item recalling intervention just administered), we modified the tense of the Pre-TEQ questions and administered at the end of each respective active PoC prompt condition (i.e., Post-TEQ) as recommended by (28). The benchmark of ≥ 70% acceptability (defined as a rating of “Very” or “Totally”) was also used as a criterion of success.

To further assess acceptability and inform future intervention development, additional pragmatic questions were developed by the research team and assessed following each active PoC prompt condition. Participants were asked to rate the general usefulness and frequency usefulness of the prompts, for both active PoC conditions. Furthermore, desired frequency (i.e., more or less frequently than the ~eight prompts sent/day) and time of day (i.e., morning, afternoon, and no preference) for prompts were also assessed. Participants were also asked to recall how often they received the prompts (i.e., “approximately a few times per day (i.e., two or three),” “approximately five to seven times per day,” “approximately eight times per day,” “greater than eight times per day, “Never,” and “I don't recall”), how frequently they noticed the prompt messages appear on their computer screen, and how frequently they responded to the prompts by standing (7-point Likert scale from “none of the time” to “all of the time”). Lastly, receipt of the prompts was objectively assessed by extracting open rates from MailChimp.

### Statistical analysis

Demographic data were summarized using means, standard deviations, frequencies, and percentages. To assess preliminary efficacy, the independent variable was condition (i.e., no-prompt control vs. TD-PoC vs. R-PoC) and the dependent variables were activPAL-measured sedentary time variables. Mixed-effects linear regression models for change were used to account for clustering of observations within participants and to determine if there were significant differences in sedentary behaviors across the three conditions. All models were adjusted for age, race, ethnicity, gender, job type, condition period (i.e., no-prompt control, R-PoC, or TD-PoC), and condition order (i.e., randomization). To assess preference and acceptability, item-level, overall score, and percent rating “Very” and “Totally” responses were summarized. McNemar tests were performed to assess between condition differences in the percent ratings. All analyses were performed using IBM SPSS Statistical Package SPSS software version 24 (IBM Analytics).

## Results

### Study flow and participant characteristics

Figure 1 presents the flow of screened and enrolled participants. A total of 41 individuals were eligible for the study with 18 ineligible (i.e., did not meet inclusion criteria, did not respond, or no interest). Twenty-three participants consented to participate; of those, four were no longer interested prior to starting the study protocol. A total of 19 participants started the trial and all completed the entire protocol. Participants reported having their sit-stand workstations for 13.1 ± 12.0 months (1 month – 48 months) prior to starting the study. Table 2 presents demographic characteristics and Table 3 presents objectively measured workplace sedentary and more active behaviors during the no-prompt control, basic reminder, and theory-driven conditions. In general, this sample of office workers consisted of primarily middle-aged Caucasian women who had sit-stand workstations for a minimum of 1 month prior to participation. Overall, participants had 3.6 ± 1.1 valid days with >4 h of activPAL wear time at work.

**Table 2:** Participant characteristics.

	**M ± SD/(frequency)%**
Age, M ± SD	39.4 ±10.74
Female	15 (78.9)
**RACE/ETHNICITY**	
Non-hispanic white	15 (78.9)
Hispanic	2 (10.5)
Black	0 (0.0)
Asian	1 (5.3)
Other	1 (5.3)
**JOB TYPE**	
Executive	4 (21.1)
Professional	7 (36.8)
Clerical	8 (42.1)

**Table 3:** Workplace sedentary behaviors during the No-prompt control, Theory-driven PoC, and Basic Reminder PoC conditions.

	**No-prompt control**		**Basic reminder PoC**		**Theory-driven PoC**	
	**M**	**(SD)**	**M**	**(SD)**	**M**	**(SD)**
Sitting	267.9	(68.0)	251.3	(86.8)	255.5	(77.7)
Standing	170.2	(69.3)	193.2	(85.8)	185.6	(77.3)
Sit-stand transitions^*^	5.9	(2.1)	6.6	(3.0)	6.3	(2.2)
Sit bouts >30 min	128.7	(70.3)	106.0	(74.7)	99.5	(59.6)
Total stepping time	41.9	(16.6)	35.5	(13.2)	39.0	(8.7)

Sitting, standing, sit bouts >30 min, and total stepping time (LPA+MVPA) are in minutes and standardized to an 8 h workday.

* Sit-stand transitions are expressed as an average average per sedentary hour. LPA, light-intensity physical activity (<100 steps per minute); MVPA, moderate-vigorous physical activity (>100 steps per minute)

### Workplace sedentary behavior

**Figure 2:**
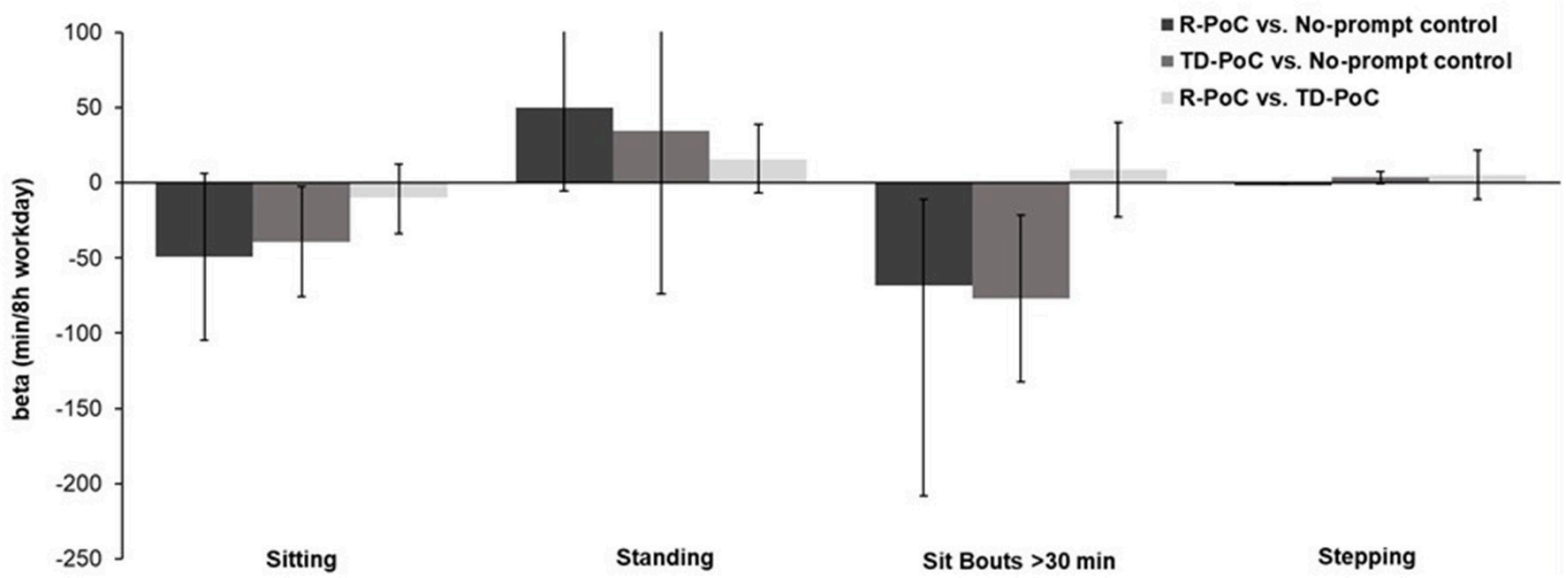
Condition effects on sedentary behavior outcomes. Values are unstandardized beta coefficients and associated 95% confidence intervals.

As displayed in Table 4 and Figure 2, sitting time was significantly lower and standing time was significantly higher for the R-PoC prompt condition relative to no-prompt control. In addition, for the R-PoC prompt condition, the number of sit-stand transitions per sedentary hour was also significantly higher relative to no-prompt control. However, during both the R-PoC prompt and TD-PoC prompt conditions, time spent in prolonged sit bouts (>30 min in duration) significantly decreased relative to the no-prompt control. Total stepping time did not change for either study condition relative to the no-prompt control. For comparisons between the active study conditions (i.e., R-PoC vs. TD-PoC), no significant differences were observed, although as shown in Figure 2, there was a trending pattern toward more favorable results for TD-PoC prompts relative to the R-PoC prompt. Both active conditions reduced sitting time and increased standing time by >30 min/day relative to no-prompt control.

**Table 4:** Mixed-effects regression outcomes by study condition.

	**Basic reminder PoC prompts vs. No prompt control**			**Theory-driven PoC prompts vs. No prompt control**			**Basic reminder PoC prompts vs. theory-driven PoC prompts**		
	**Beta**	**(SE)**	***P***	**Beta**	**(SE)**	***P***	**Beta**	**(SE)**	***P***
Sitting	**-49.0**	**(20.8)**	**0.03**	−39.1	(20.4)	0.06	−9.9	(11.6)	0.40
Standing	**49.8**	**(19.7)**	**0.02**	34.3	(19.4)	0.09	15.5	(11.0)	0.17
Sit-stand transitions	**2.3**	**(1.1)**	**0.04**	2.0	(1.0)	0.06	−0.3	(0.6)	0.62
Sit bouts >30 min	**-68.1**	**(27.8)**	**0.02**	**-76.7**	**(27.1)**	**0.01**	8.6	(15.4)	0.58
Total stepping time	−1.7	(5.2)	0.75	3.6	(5.1)	0.49	5.2	(2.9)	0.08

*Significant results are bolded. All models were adjusted for age, gender, race/ethnicity, job type, order, and period*.

### Preference of PoC prompts

Table 5 presents preference metrics for both the active PoC prompt conditions (assessed prior to intervention delivery). While the R-PoC prompt condition met the benchmark for the “Easy” construct only, the TD-PoC prompt condition met the 70% benchmark for the following constructs: “Logical,” “Appropriate,” “Recommend,” and “Availability.” No significant differences were observed for any items in reaching the 70% benchmark for preference.

**Table 5:** Point-of-choice study conditions preference.

	**Preference (Pre-TEQ)**			
	**Basic reminder PoC prompt**		**Theory-driven PoC prompts**	
	**M (SD)**	**Benchmark** >**70%**	**M(SD)**	**Benchmark** >**70%**
Logical	3.1 (0.3)	66.7	3.4 (0.1)	**93.8**
Easy	2.9 (0.3)	**73.7**	2.9 (0.1)	68.8
Appropriate	2.8 (0.4)	63.2	3.3 (0.3)	**87.5**
Helpful	2.5 (0.6)	52.6	2.8 (0.1)	68.8
Successful	2.3 (0.3)	47.4	2.7 (0.1)	62.5
Confident	2.8 (0.3)	68.4	2.6 (0.1)	56.3
Recommend	2.7 (0.5)	68.4	3.3 (0.1)	**93.8**
Availability	2.5 (0.4)	52.6	3.1 (0.1)	**87.5**
Total TEQ Score	2.7 (0.4)	61.6	3.0 (0.1)	**77.3**

*Item ranges were all 1–5. Preference was assessed prior to intervention delivery. Preference score meeting the >70% success criterion are bolded. All pairwise comparisons between basic reminder PoC prompt vs. theory-drive PoC prompt and pre TEQ vs. post TEQ were non-significant (p's > 0.05)*.

### Acceptability of PoC prompts

Table 6 presents acceptability metrics for both the active PoC prompt conditions (assessed post intervention delivery). Both the TD-PoC and R-PoC prompt condition met the >70% benchmark for the “Logical” and “Recommend” constructs; however, the TD-PoC prompt condition also met the 70% benchmark for “Availability.” Regarding additional acceptability outcomes also assessed post intervention delivery, slightly less than half of the participants rated the PoC prompts as “Very” or “Extremely useful” (i.e., “General Usefulness”) in helping them to stand more with their sit-stand workstation for both PoC prompt conditions. Similar findings were seen for the frequency of the PoC prompts being useful for increased standing (i.e., “Frequency Usefulness”). Approximately half of participants rated the R-PoC prompt “Time to STAND!” as “Very Useful” or “Extremely Useful”. The TD-PoC self-efficacy based prompts were only rated by a quarter of participants as “Very Useful” or “Extremely Useful”; however, more moderate ratings were displayed for the outcome expectancy based prompts, and the proximal goal based prompts, with the latter being rated the highest for both conditions. For both the R-PoC and TD-PoC prompt conditions, the majority of participants reported no time of day preference for receiving prompts however, fewer than half of R-PoC and TD-PoC participants reported that they would prefer to receive prompts in the afternoon, and <5% of R-PoC and TD-PoC participants reported wanting to receive the prompts in the morning. A high percentage of participants recalled receiving both the R-PoC and TD-PoC prompts 5–8 times/day and the vast majority reported seeing the prompts appear on their computer screen 50% or more of the time. In addition, data from MailChimp revealed that the vast majority of participants received at least seven prompts per day.

**Table 6:** Point-of-choice study conditions acceptability.

	**Basic reminder PoC prompt**		**Theory-driven PoC prompts**	
	**M(SD)**	**Benchmark >70%**	**M (SD)**	**Benchmark >70%**
Logical	3.3 (0.4)	**84.2**	3.1 (0.3)	**73.7**
Easy	2.6 (0.2)	63.2	2.7 (0.0)	68.4
Appropriate	3.1 (0.5)	68.4	2.9 (0.3)	68.4
Helpful	2.4 (0.6)	47.4	2.5 (0.4)	63.2
Successful	2.2 (0.6)	47.4	2.2 (0.1)	47.4
Confident	2.7 (0.3)	63.2	2.8 (0.2)	68.4
Recommend	3.0 (0.4)	**73.7**	2.9 (0.3)	**72.2**
Availability	2.6 (0.4)	57.9	2.8 (0.3)	**73.7**
Total TEQ Score	2.7 (0.4)	63.2	2.7 (0.2)	68.9
General usefulness	2.2 (1.3)	42.1	3.7 (1.7)	47.4
Frequency usefulness	2.3 (1.3)	47.4	2.3 (1.2)	42.1
Reminder usefulness	2.3(1.3)	52.6	–	–
SE usefulness	–	–	1.5 (1.3)	26.3
OE usefulness	–	–	2.2 (1.1)	42.1
PG usefulness	–	–	2.4 (1.2)	63.2

*All acceptability ranges were 0-4; SE, self-efficacy; OE, outcome expectations; PG = proximal goal; Acceptability was assessed following intervention delivery. Acceptability scores meeting the >70% success criteria are bolded. All pairwise comparisons between basic reminder PoC prompt vs. theory-driven PoC prompts were non-significant (p's > 0.05)*.

## Discussion

The purpose of this study was to examine the preliminary efficacy, preference, and acceptability, of two PoC prompt interventions relative to no-prompt control for reducing sedentary behaviors in office workers with suboptimal utilization of their sit-stand workstations. Both prompt types appeared efficacious for reducing sedentary time and increasing standing time overall, a slight trend appeared toward greater efficacy for the R-PoC prompt condition for total sitting time, and the TD-PoC prompt condition for reducing prolonged bouts of sitting. While no significant differences between the two PoC prompt conditions were observed, the effect sizes provide both content and temporal insights that may inform future workplace sedentary behavior reduction PoC Overall, preference was slightly better for TD-PoC than R-PoC and acceptability met the 70% benchmark for some but not all metrics for TD-PoC and R-PoC prompt conditions.

### Efficacy of point-of-choice prompts in sit-stand workstation users

Similar to previous findings, the reminder prompts which used repetitive content “Time to STAND!,” resulted in a significant decrease in sitting time and increase in standing time relative to no-prompt control (22, 26, 38). However, contrary to our hypotheses, TD-PoC prompts only elicited significant reductions in prolonged sitting bouts (>30 min of continuous sitting) relative to the no-prompt control. Further, the TD-PoC prompts and were less efficacious than basic reminders for sitting and standing time outcomes. These contradictory findings may be attributed to differences in prompts dosage, sample population, study duration, PoC prompt content, and specificity of behavioral targets.

#### Point-of-choice prompt dosage and duration

While the duration of this study was the same as Evans et al. (26), prompt dosage (i.e., once every 30-min for 1 min) was higher than ours, and this may have implications for efficacy of a higher prompt dose needed to elicit greater reductions in total sitting time as well as prolonged sit bouts. Similarly, the lower average daily sedentary time (measured during the no-prompt control condition) in our study may have contributed to these conflicting findings. However, it is important to note that the reduction in total sitting time was similar between conditions. In addition, the prompting software used by Evans et al. (26) maintained on participants computer screens for 1 min, whereas the prompts in the present study and others (22) could easily be missed if not looking directly at one's computer screen. Therefore, it may be necessary to have prompts appear for a minimum amount of time to properly serve as a visual cue. Additionally, a higher PoC prompt dose may prove to be beneficial considering literature displaying breaks in prolonged sitting time being beneficial for metabolic risk and it may be of necessity given the habitual nature of sedentary behaviors.

It is difficult to judge whether duration of the intervention may have impacted the results, though past research has shown decreases in sitting time during brief 3-day (25) and 5-day (26) intervention periods. Longer duration interventions and studies with follow-up periods may provide insight into whether PoC prompts are needed for long-term sustain standing behaviors, or if there is a minimum time-period required to elicit independent behavior change. Conducting longer duration interventions may provide insight as to whether there is a trend for office-workers to return to habitual sitting durations after the removal of prompts and are of important consideration for fostering behavior change that can be sustained independently.

#### PoC prompt content and specificity of behavioral targets

This study focused on the PoC prompts as a complementary strategy to facilitate sit-stand workstation use, which is conducive to standing behavior. The contextual specificity of the reminder prompt (“Time to STAND!”) and behavioral target (standing) may partially account for the increased standing time and notably, the significant decrease in prolonged sit bouts (≥30 min). In contrast, the TD-PoC prompts included a broader range of messages (i.e., 40 unique prompts) that encompassed behavior change tactics from the social cognitive theory (i.e., self-efficacy, outcome expectations, and proximal goal setting) that largely focused on not only standing more, but the importance of reducing prolonged sitting bouts. In support of the “usefulness” results (Table 4), the significant decrease in prolonged sit bouts observed for the TD-PoC condition compared to no-prompt control, may be largely driven by the perceived consequences of performing or not performing sedentary behaviors (i.e., outcome expectations) and behavioral targets (i.e., proximal goal setting), designed to foster active behaviors while working. These findings may further strengthen the argument for developing and implementing tailored interventions that leverage specific types of prompts (e.g., basic reminder vs. theory-driven health outcome specific) to focus on different aspects of sedentary behaviors (e.g., standing vs. reduced prolonged sitting bouts).

### Implications of preference and acceptability of PoC prompt findings

#### Implications for PoC prompt content

Prior to the intervention, participants reported preferring TD-PoC prompt content compared to basic reminders. As preference may be used as an early indicator of intervention adoption, this may suggest greater chances for cultivating sustained behavior change (28). The use of multiple observations (i.e., pre and post) throughout the present study duration of future PoC based studies allows for richer examination of fluctuations in prompt content preference.

The TD-PoC prompt content was also rated as more acceptable than the basic R-PoC prompt content, replicating trends observed for preference. However, the trajectory of the TEQ results from pre-to-post indicated a ~10% decline in the TD-PoC content due to lower acceptability ratings, whereas the reminder content displayed a marginal increase (i.e., ~2%) from pre-to-post TEQ scores. We postulate that the trend for the interventions to be rated more similarly at post-test may be due an initial perception of the “unique” theory-driven intervention being perceived as more efficacious than one including basic “repetitive” reminder prompts. However, post-intervention delivery, participants may have concluded that a basic reminder may be sufficient for prompting standing behavior. Furthermore, these results were supported by the additional acceptability questions, beyond the TEQ measure, which exhibited similar perceived usefulness ratings across both conditions also assessed following each active condition.

Interestingly, for individual ratings of the TD-PoC prompt content post condition delivery, proximal goals were perceived as most useful and self-efficacy content was perceived as least useful. We posit that due to the habitual nature of sitting and simplicity of the intervention behavioral target (moving from a seated to standing position with an existing sit-stand workstation), self-efficacy prompt content may not be necessary to facilitate sit-stand transitions beyond the start of the intervention. Alternatively, setting proximal goals to guide sit-stand workstation use over time may be more valuable for long term behavior change. Consequently, prompt content may need to evolve over the intervention delivery period, including both TD-PoC content alongside regular reminders at the start of the intervention, and over time, transitioning to basic reminders only.

#### Implications for temporal decisions regarding PoC prompts

Across both prompt conditions, the additional acceptability assessment post condition delivery revealed that about half of the participants were least receptive to receiving prompts in the morning and most receptive to receiving prompts in the afternoon. This is consistent with anecdotal reports of participants indicating that persons are more motivated to stand in the morning; however, fatigue may inhibit standing as the day goes on (16). Alternatively, nearly 50% of the participants reported “no preference” regarding the time of day. Given the habitual nature of sitting, it may be difficult for participants to “recollect” the time(s) at which they are likely to sit for prolonged periods. Further examination of workplace sedentary time trajectories is required to better understand when prompts may be most effective, considering both opportunity and receptivity.

Further assessment post condition delivery revealed that most participants reported that the frequency of the TD-PoC prompts was appropriate compared to the reminder prompts. Interestingly, a higher proportion of participants reported that they would prefer to receive prompts more frequently for both the R-PoC and TD-PoC conditions. These results support a likely need for tailored interventions that may incorporate real-time feedback whenever possible to promote behavior change (39). Researchers can use such real-time feedback to detect prolonged bouts of sitting, which are of particular importance in workplace settings where there are greater opportunities. Individuals may be more receptive to these types of interventions given variations in busyness and workload are likely. Automatic detection of prolonged sitting may also overcome the potential for negative feedback of prompting someone to stand when they are already standing.

### Strengths and limitations

Strengths of our study include the randomized cross-over trial design and 1-week wash-out period, providing a balance between the R-PoC and TD-PoC prompt conditions and reducing potential carry-over effects between the two interventions. Participants also had their sit-stand workstation for at least 1 month (range of 1–48 months) prior to starting the intervention, possibly minimizing workstation specific novelty effects. Objective measures of sedentary and more active behaviors were assessed. This study also utilized objective measures of prompt engagement (i.e., delivery and open rates), allowing for implementation measurement. Finally, other studies (25, 26, 38, 40) have generally examined the effectiveness of interventions without assessing preferences and acceptability, potentially limiting the ability to achieve important knowledge that can further enhance, develop, and establish an intervention as evidence-based for future implementation and dissemination. Assessing these factors is important for the development of evidence-based interventions and to help researchers to understand the probability of an intervention being efficacious (36). Limitations of this study include relatively sample size. Participants in this study were mostly university staff, primarily women, who volunteered to participate and therefore may not be representative of other non-volunteer office workers. Also, this was a short-term study, so longer efficacy of PoC prompts in this context remains unknown.

### Future directions

Future studies further examining the preliminary efficacy, preference, or acceptability of using prompts to increase sit-stand workstation utilization should consider the below adaptations to enhance study design.

- Larger sample size
- Recruitment across sectors (i.e., government, industry, academia)
- Feedback and integration with worksite wellness personnel
- Education on health implications
- Incorporation of real-time feedback (i.e., wearable devices and proximity sensors)
- Prompt delivery modality (i.e., computer software, email, or text-messaging)
- Determination of optimal prompt dosage/day

## Conclusion

This study highlights the potential efficacy of utilizing point-of-choice prompts to reduce sedentary behaviors in desk-based office workers with suboptimal reported sit-stand workstation usage. While a basic reminder prompt may be sufficient to elicit reductions in sedentary behaviors during this short-term study, theory-driven point of choice prompts may prove to be beneficial for driving reductions in specific behaviors sedentary behaviors (e.g., reduced sitting time vs. reduced time spent in prolonged sitting bouts). Interestingly, despite the theory-driven point of choice prompt condition having higher total preference and acceptability scores, the basic reminder point of choice prompt condition displayed a significant reduction in sitting time and significant increase in standing time compared to the theory-driven point of choice prompt condition.

## Author contributions

All authors contributed to study design. ML was responsible for data collection, analysis, and manuscript preparation. SM, MT, MP, JH, BA, and MB reviewed and edited the manuscript, and approved the final version prior to submission.

### Conflict of interest statement

The authors declare that the research was conducted in the absence of any commercial or financial relationships that could be construed as a potential conflict of interest.
